# Human breast milk as source of sphingolipids for newborns: comparison with infant formulas and commercial cow’s milk

**DOI:** 10.1186/s12967-020-02641-0

**Published:** 2020-12-14

**Authors:** Michele Dei Cas, Rita Paroni, Paola Signorelli, Alessandra Mirarchi, Laura Cerquiglini, Stefania Troiani, Samuela Cataldi, Michela Codini, Tommaso Beccari, Riccardo Ghidoni, Elisabetta Albi

**Affiliations:** 1 Department of Health Sciences, Università degli Studi di Milano, Milan, 20142 Italy; 2 Department of Pharmaceutical Sciences, University of Perugia, Perugia, 06126 Italy; 3 Struttura Complessa di Neonatologia e Terapia Intensiva Neonatale– Azienda Ospedaliera Santa Maria della Misericordia, Perugia, 06126 Italy; 4 Aldo Ravelli Center for Neurotechnology and Experimental Brain Therapeutics, Department of Health Sciences, Università degli Studi di Milano, Milan, 20142 Italy

**Keywords:** Sphingomyelin, Ceramide, Lipidomic, Human breast milk, Infant formulas, Cow’s milk

## Abstract

**Background:**

In the past two decades, sphingolipids have become increasingly appreciated as bioactive molecules playing important roles in a wide array of pathophysiology mechanisms. Despite advances in the field, sphingolipids as nutrients remain little explored. Today the research is starting to move towards the study of the sphingomyelin content in human breast milk, recommended for feeding infants.

**Methods:**

In the present study, we performed a lipidomic analysis in human breast milk in relation with maternal diet during pregnancy, in infant formulas, and in commercial whole and semi-skimmed milks for adults. Mediterranean, carnivorous and vegetarian diets were considered.

**Results:**

The results showed that total sphingomyelin, ceramide and dihydroceramide species are independent on the diet. Interestingly, the milk sphingolipid composition is species-specific. In fact, infant formulas and commercial milks for adults have a lower level of total sphingomyelin and ceramide content than human breast milk with very different composition of each sphingolipid species.

**Conclusions:**

We conclude that human breast milk is a better source of sphingolipids than infant formulas for baby nutrition with potential implications for the brain development and cognitive functions.

## Background

Despite extensive basic research on the role of sphingolipids (Sphs) in brain maturation and pathophysiology, very little attention is still paid today to Sphs as nutrients.

### Sphingolipid metabolism

**Fig. 1:**
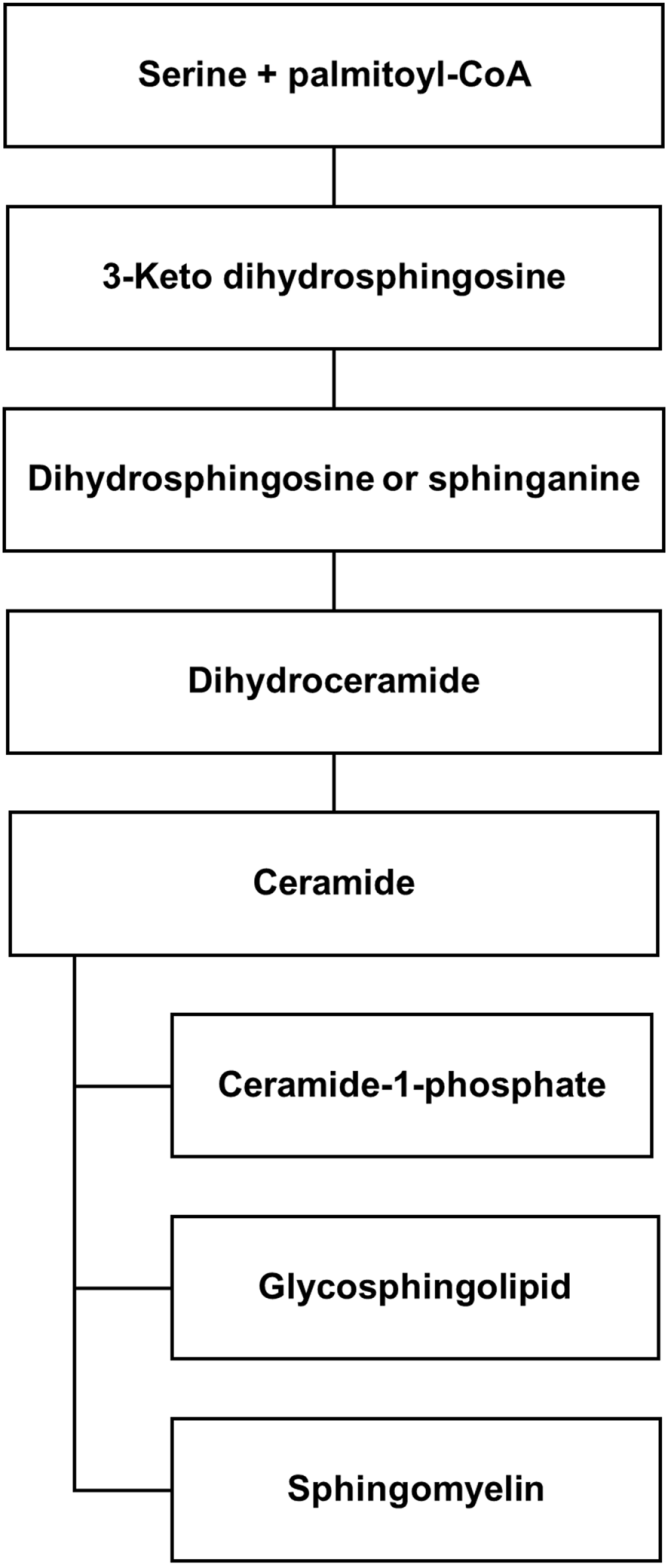
Diagram of sphingolipid biosynthetic pathways

Sphs are a family of bioactive molecules metabolically interconnected with each other due to many enzymes that generate, catabolize, or interconvert them [1]. The key molecules of this complex metabolic machine are ceramide (Cer) and sphingomyelin (SM) (Fig. 1). De novo synthesis of Cer occurs in the Golgi apparatus and starts from the serine and palmitoyl-CoA condensation to produce 3-ketodihydrosphingosine, then reduced to dihydrosphingosine or sphinganine. Cer-synthase N-acylates the sphinganine to produce dihydroceramide (DHCer) that is desaturated by dihydroceramide desaturase to generate Cer [1]. Depending on the different Cer-synthase isoforms, from 1 to 6, Cers with different acyl chain lengths can be produced [2]. Cers can be phosphorylated to Cer-1-phosphate, can be converted to glycosphingolipid (GLS) or can be used to synthesize SM by SM-synthase that uses phosphatidylcholine as donor of phosphocholine group [3]. In turn, SM can be hydrolyzed by five sphingomyelinases, located in different subcellular fractions and activated at different pH, to produce Cer [3]. Cers produced from SM and GSL degradation in lysosomes are exported from these through specific proteins to reach again the Golgi apparatus where they are recycled and re-utilized in salvage processes [4].

### Sphingolipid functions

SMs and Cers are implicated in multiple cellular functions, including proliferation, differentiation, apoptosis, inflammation and cancer with different mechanisms of action. In particular, to regulate cell fate SMs interact with cholesterol to form lipid rafts that also contain Cers [5, 6]. In cell membrane, lipid rafts act as platform for hormonal and growth factor receptors, and are involved in cell signaling [7–10]. In nuclear membrane, they act as platform for cell signaling molecules [11, 12].

### Role in brain development

In the brain system, SM is an essential component of myelin that surrounds neuronal axons [13]. The low intake with diet of essential lipids for SM synthesis, i.e. nervonic acid, increases demyelination and reduces the remyelination process [14]. Moreover, changes of SM species are associate with different brain pathologies [15, 16]. The role of SM in brain disorders is due to its involvement in the myelination process and, therefore, in the axonal maturation [17]. Considering that myelination is closely associated with the maturation of brain networks, the coordination of information processing and in the cognitive performance [18], the nutrition is relevant in infants and children [19]. However, the role Sphs in growth is not limited to their effect on the central nervous system.

### Role in immune system

It has been demonstrated that Sphs are particularly relevant in development and regeneration of different cells and tissues. Notably, sphingosine-1-phosphate (S1P) is a major chemoattractant responsible for stimulation of egress of bone marrow-derived immune and stem cells [20] with involvement of radical oxygen species signaling and stromal cell-derived factor 1 release [21]. Also, various studies demonstrated that S1P content in human adipose tissue, both subcutaneous and visceral, is also associated with levels of various pre-regenerative and immune substances [22, 23]. Therefore, Sphs play a major role in development of proper immune system as well as regeneration/development of solid organs.

### Sphingolipids in human milk

Few studies have currently been performed on the content of SM in human breast milk (HBM). It has been demonstrated that SM is present in HBM and represents about 37% of the total phospholipid fraction [24]. A more recent study indicated that mature breast milk contains a high variability of SM content [25] and it seems be linked to the gestational age. In fact, both gestational age and lactational age affect SM content, together to other lipids, in HBM; in particular, preterm HBM contains higher level of total SM and SM species than full term milk in colostrum and transitional milk [26]. In this case, the high level of SM and other lipids is associated with faster growth of preterm infant [27]. These studies give a picture of the variability of SM in HBM, although extensive evidence to support this claim is lacking, especially in reference to the maternal diet. Likewise, many studies have not analyzed yet the level of Cer, a molecule that represents both the structural basis for the synthesis of SM and its first catabolic product. Therefore, despite findings that SM species are present in high content in HBM, no data about Cers in HBM were reported.

The aim of the work was to investigate: (1) SMs, Cers, and DHCers in HBM in relation with Mediterranean diet (MD), vegetarian diet (VD), and carnivorous diet (CD); (2) the differences in the milk sphingolipid composition between mothers fed on MD with preterm birth (PTB) and mothers fed on MD with full term birth (FTB); (3) differences between infant formulas (Fs) and HBM of mothers fed on MD; (4) sphingolipid composition in whole and semi-skimmed cow’s milks for adults. The results show that total SM, Cer, and DHCer species in HBM are independent of both the diet and on gestational age. Fs and cow’s milks for adults are very different in sphingolipid composition from HBM, by suggesting that milk sphingolipid composition might be species-specific.

## Methods

### Reagent and chemicals

Lipids standards were purchased from Avanti Polar Lipids (Alabaster, AL, USA). The chemicals, at analytical grade, were purchased by Sigma-Aldrich (St. Louis, MO, USA). All aqueous solutions were prepared using purified water at a Milli-Q grade (Burlington, MA, USA).

### Population

Twentyfour milk donor women at *Banca del Latte* Umano Donato (BLUD, Struttura Complessa di Neonatologia e Terapia Intensiva Neonatale– Azienda Ospedaliera Santa Maria della Misericordia - Perugia, Italy) were considered for the the study from April 2019 to October 2019. The project was approved by the Bioethics Committee of Perugia University (n.2018-05) and all procedures were performed accordingly. All donors’ participation was on a voluntary basis and signed participation informed consent. Donors were anonymized and no sensitive data were collected. Inclusion criteria were mothers considered as good milk donors for the absence of pathologies transmissible with milk at different age, and with body mass index (BMI) > 25 and BMI < 25 at the beginning of pregnancy. The exclusion criteria were mothers with a history of pathology, and users of drug, alcool and smoke. Therefore, 1 donor (n°14) was excluded because of type 2 diabetes and severe hypothyroidism.

Mothers were invited to answer questions in a questionnaire that had the aim of knowing: (1) the age; (2) the weight before pregnancy; (3) weight gain during pregnancy; (4) weight gain at the time of the study compared to weight before pregnancy; (5) gestational time and weight of the newborn; (6) nutritional habits of the mothers during pregnancy and during breastfeeding by distinguishing Mediterranean diet (MD, n = 15) or diet including animal and vegetable products, carnivorous diet (CD, n = 4) or diet very rich in meat and fish with very low level of carbohydrates and without fruit and vegetables, and vegetarian diet (VD, n = 4) or diet including only vegetable products.

Five mothers (22%) gave preterm birth (PTB) and eighteen (78%) had full term birth (FTB).

### Milk samples

For the study only samples of mature milk, collected on the 10th day of lactation, were considered. Immediately after collecting, the milk samples were submitted to Holder pasteurization that aims to rid milk of potentially harmful germs by heating it to 62.5 °C (145 °F) for half an hour, and then cooling it back down to 4-10 °C, as previously reported [28]. The Fs were all cowmilk-based and were prepared in accordance with the instructions of the manufacturing companies. All samples were then stored in a − 20 °C freezer before analysis. Homogenized-pasteurized cow’s milk was obtained in groceries and consisted of whole milk (n.4) with fat content ranging from 3.2–3.6%, whereas semi-skimmed milk (n.2) contains 1.6–2.0% in fats.

### Lipid extraction and targeted sphingolipid analysis

Sphingolipid extraction and LC–MS/MS analysis were performed as previously described [29]. Sphs were extracted from three independent 25 µL aliquots of HBM, commercial Fs (reconstituted according to the manufacturer instruction) or bovine milk, using a monophasic solvent extraction (chloroform/methanol/water, 30:60:10, v/v/v). They were analyzed by LC (Dionex 3000 UltiMate,ThermoFisher Scientific) coupled to a tandem mass spectrometer (AB Sciex 3200 QTRAP, Sciex). The separation was achieved by a reversed-phase analytical column (Acquity BEH C8 100 × 2.1 mm × 1.7 μm, Waters) through a linear gradient between eluent A (0.2% formic acid, 2 mM ammonium formate water-solution) and eluent B (0.2% formic acid, 1 mM ammonium formate in methanol). Quantitative analysis was performed interpolating each peak area of analyte/area internal standards with a calibration curve for each sphingolipid. Experimental details were reported in Additional file 1: Table S1.

### Statistical analysis

To study correlation between different parameters of population, Pearson’s correlation coefficient test was performed by SPSS program. Statistical analysis of lipidomic study was performed with GraphPad Prism 7.0 (GraphPad Software, Inc, La Jolla, CA, USA). In particular, statistical differences were investigated by either unpaired t test or one-way ANOVA coupled with Bonferroni post hoc test, in the case of more than two experimental groups. Graphs were represented as mean ± SD, and statistical significance was set as follows *p < 0.05; **p < 0.01; ***p < 0.001; ****p < 0.0001.

Multivariate analysis was completed by MetaboAnalyst server (version 4.0). Data were checked for integrity, filtered by interquartile range, log-transformed (generalized logarithmic transformation), and auto-scaled prior to statistical analysis. The supervised multivariate classification was achieved by partial least squares-discriminant analysis (PLSDA) and hierarchical clustering coupled with heatmap. In the heatmap analysis, the clustering algorithm was set to Ward and the distance measure to Euclidean.

## Results

### Population and diet

A total of 23 milk donor mothers were included in this study since 1 donor (n°14) was excluded because of type 2 diabetes and severe hypothyroidism. From the analysis of the answers to the interview resulted that very young new mothers or new mothers of late age were not present in the study group; mothers included in the study showed a mean age of 32.5 ± 4.7 years. The weight and BMI were reported in the Table 1.

**Table 1:** Anthropometric mother parameters. Weight is expressed in kg; weight gain was calculated respect to the weight before pregnancy. In parenthesis, number of mothers who reached pre-pregnancy weight by the 10th day of lactation

	Weight before pregnancy	Number of mothers with BMI < 25	Number of mothers with BMI > 25	Weight gain after pregnancy	Weight gain at 10 days of breastfeeding
Total mothers	62.3 ±10.1	5	18	13.0 ± 4.4	9.8 ± 1.2 (5)
MD mothers	62.5 ± 9.8	4	11	13.4 ± 4.6	8.6 ± 4.7 (4)
VD mothers	64.0 ± 9.5	1	3	11.0 ± 2.3	8.3 ± 3.9 (1)
VD mothers	55.2 ± 2.6	0	4	12.8 ± 4.9	6.7 ± 5.0 (0)

Anthropometric mother parameters. Weight is expressed in kg; weight gain was calculated respect to weight before pregnancy. In parenthesis, number of mothers who restored pre-pregnancy weight by the 10th day of lactation

No significant differences in weight gain between mothers with BMI > 25 and those with BMI < 25 during pregnancy and/or lactation was evident. Pearson’s correlation coefficient test showed no correlation between age and weight before pregnancy, weight gain during pregnancy, and weight gain at the time of the study compared to weight before pregnancy. Five mothers (22%) gave preterm birth (PTB) and eighteen (78%) had full term birth (FTB). Infants born to PTB mothers had a weight of 1.77 ± 0.25 kg and those born to FTB mothers a weight of 3.28 ± 0.47 kg. The weight of infants was independent on the BMI of mothers. Four mothers suffered from hypothyroidism that arose during pregnancy and seven mothers from iron deficiency anemia. Both disorders had been kept under control with specific treatments. No significant change in terms of weight gain in pregnancy, BMI, newborn weight, PTB or FTB was found compared to controls (mothers without any change in blood count and in thyroid hormones).

Three different types of diet were considered in the questionnaire: Mediterranean diet (MD), carnivorous diet (CD), and vegetarian diet (VD). The analysis of the responses showed that of the 23 mothers studied, 15 (65%) followed an MD, 4 (17.5%) a VD and 4 (17.5%) a CD. No mom was followed by a nutritionist. The mothers who adopted the MD followed the indications of the gynecologist. The mothers who adopted VD did this out of personal choice. Mothers who adopted the CD didn’t like eating fruit and vegetables. There was no relationship between hypothyroidism/anemia and diet. In fact, 2 mothers on MD, 1 VD and 1 CD were affected by hypothyroidism and 4 mothers on MD, 2 VD and 1 CD were affected by anemia, which indicates that the insurgence of the previous diseases is independent of diet. The type of diet was not relevant for the weight gain of mothers during pregnancy (Table 1). Moreover, the weight of infants at birth was 2.67 ± 0.76 kg if mothers followed MD, 3.30 ± 0.39 kg if mothers followed VD, and 2.95 ± 0.70 kg if mothers followed CD.

### Sphs in the human breast milk

**Fig. 2:**
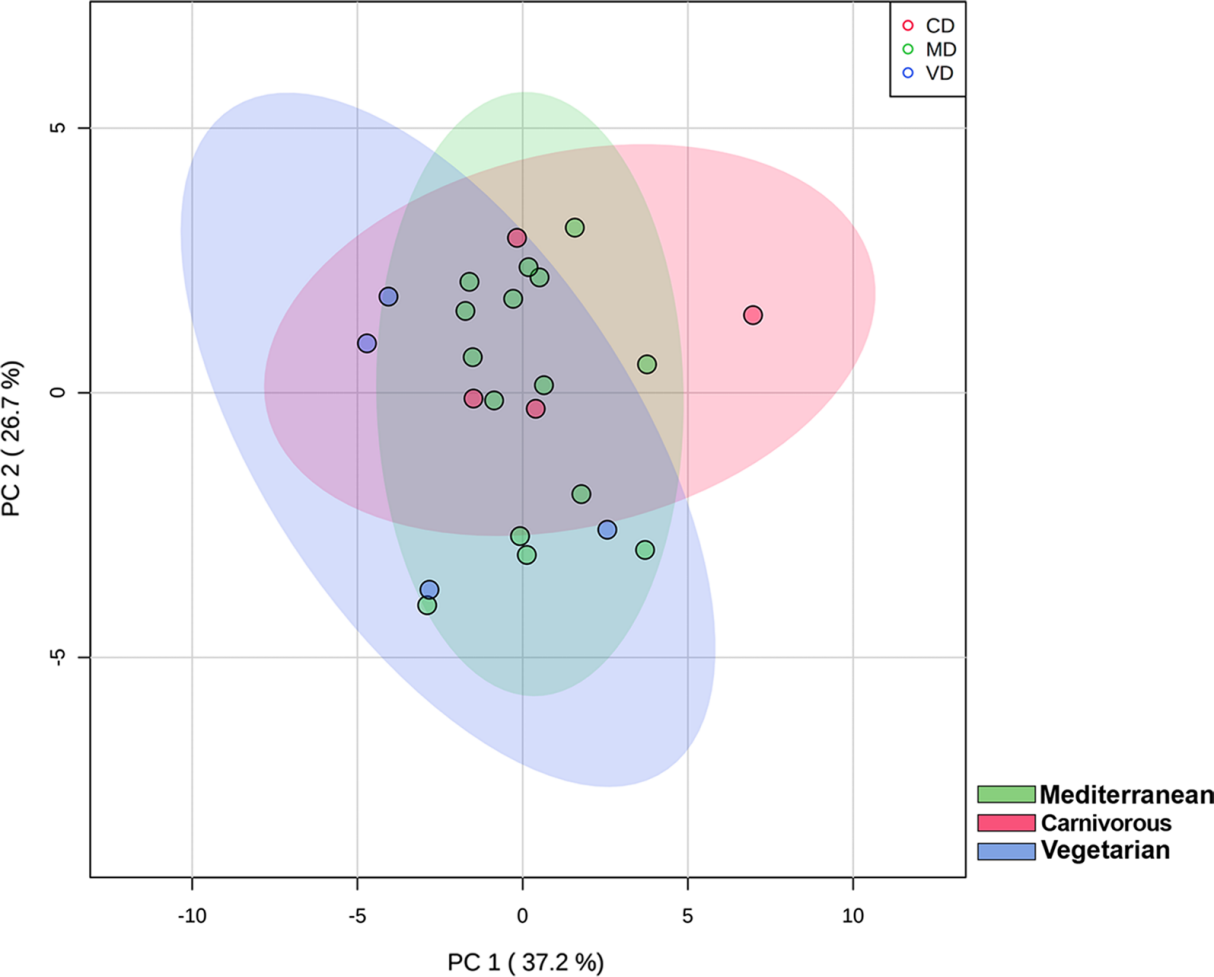
Comparison of breast milk samples according to the women’s diet by multivariate discriminant analysis (PLSDA). The axes are ranked according to their importance in the group discrimination. In the abscissa axis, component 1 (PC1, 37%) represents the maximum of the separation that can be reached within these cluster and variables, that is the direction in the original data that contains the most variance between the groups. In the ordinate axis, component 2 (PC2, 8.8%) represents the direction that contains the most remaining variance. Coloured area indicates the 95% confidence interval of each cluster

The effect of diet on the total sphingolipid content was investigated by a multivariate analysis taking in account the sphingolipid composition of the HBM samples under study. Figure 2 shows a PLSDA plot of the 23 milk samples. A significant overlap of the three clusters (carnivorous diet CD, vegetarian diet VD and Mediterranean diet MD) minimize the influence of the diet on milk sphingolipid composition.

**Fig. 3:**
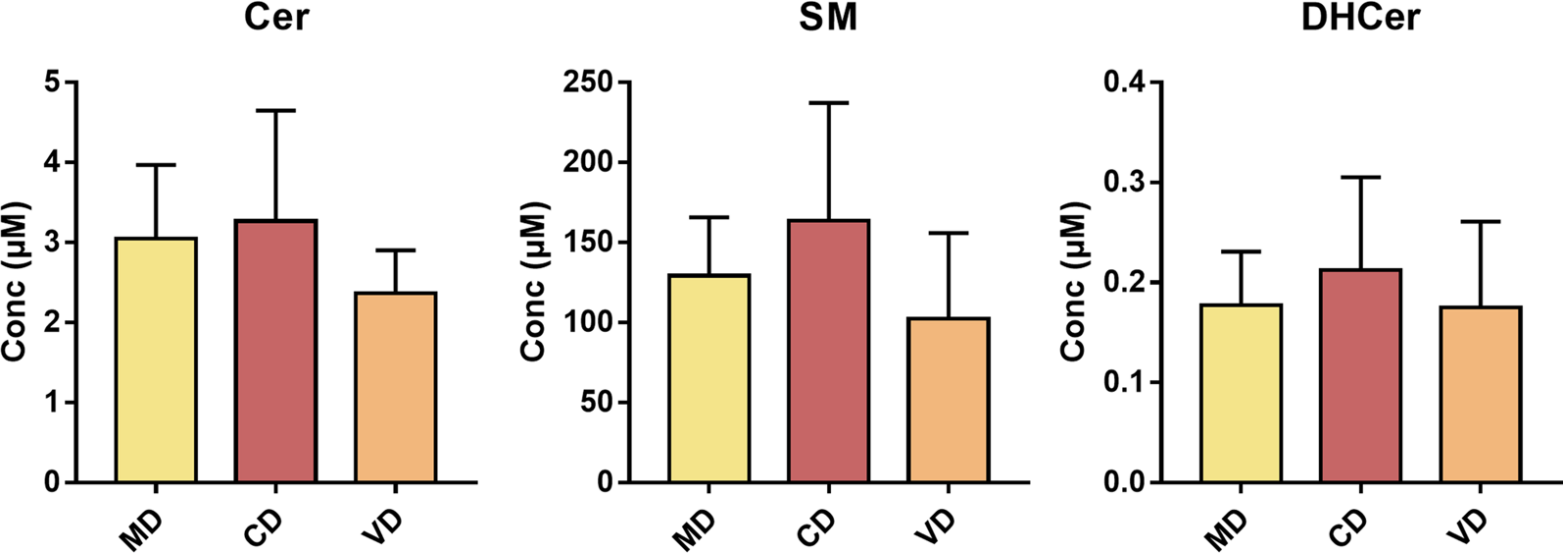
Brest milk Sphs composition depending on types of diets. Data are expressed as μM concentration and represent the mean ± SD of milk samples of mothers who followed a Mediterranean (MD, yellow, n = 15), carnivorous (CD, red, n = 4), and vegetarian (VD, orange, n = 4) diet. Cer, ceramide; SM, sphingomyelin; DHCer, dihydroceramide. No Statistical difference among groups was found by one-way ANOVA coupled with Bonferroni post hoc test

Accordingly, when you consider total Cer, SM, and DHCer (univariate analysis) separately, no significant differences are evident in relation to diet (Fig. 3).

**Fig. 4:**
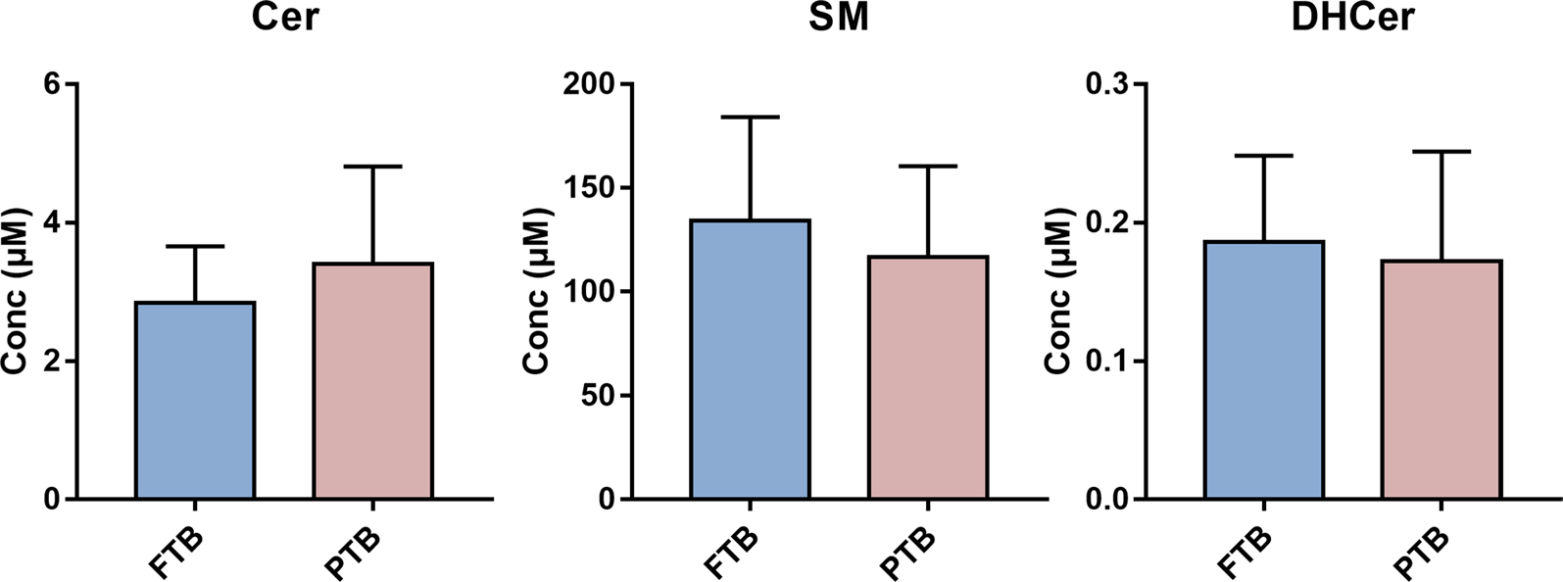
Brest milk Sphs composition depending on gestational age. Data are expressed as μM concentration and represent the mean ± SD of milk samples of mothers who had full term birth (FTB, light blue, n = 18) and of mothers who had preterm birth (PTB, pink, n = 5). Cer, ceramide; SM, sphingomyelin; DHCer, dihydroceramide. No statistical difference between groups was found by unpaired t-test

Then, we sought to investigate possible changes of sphingolipid composition in HBM in relation to the gestational age. Therefore, we analyzed the milk of mothers who had full term birth (FTB) and preterm birth (PTB). The results highlight no significant difference between the two groups (Fig. 4).

### Sphs in infant formulas and adult milks

**Fig. 5:**
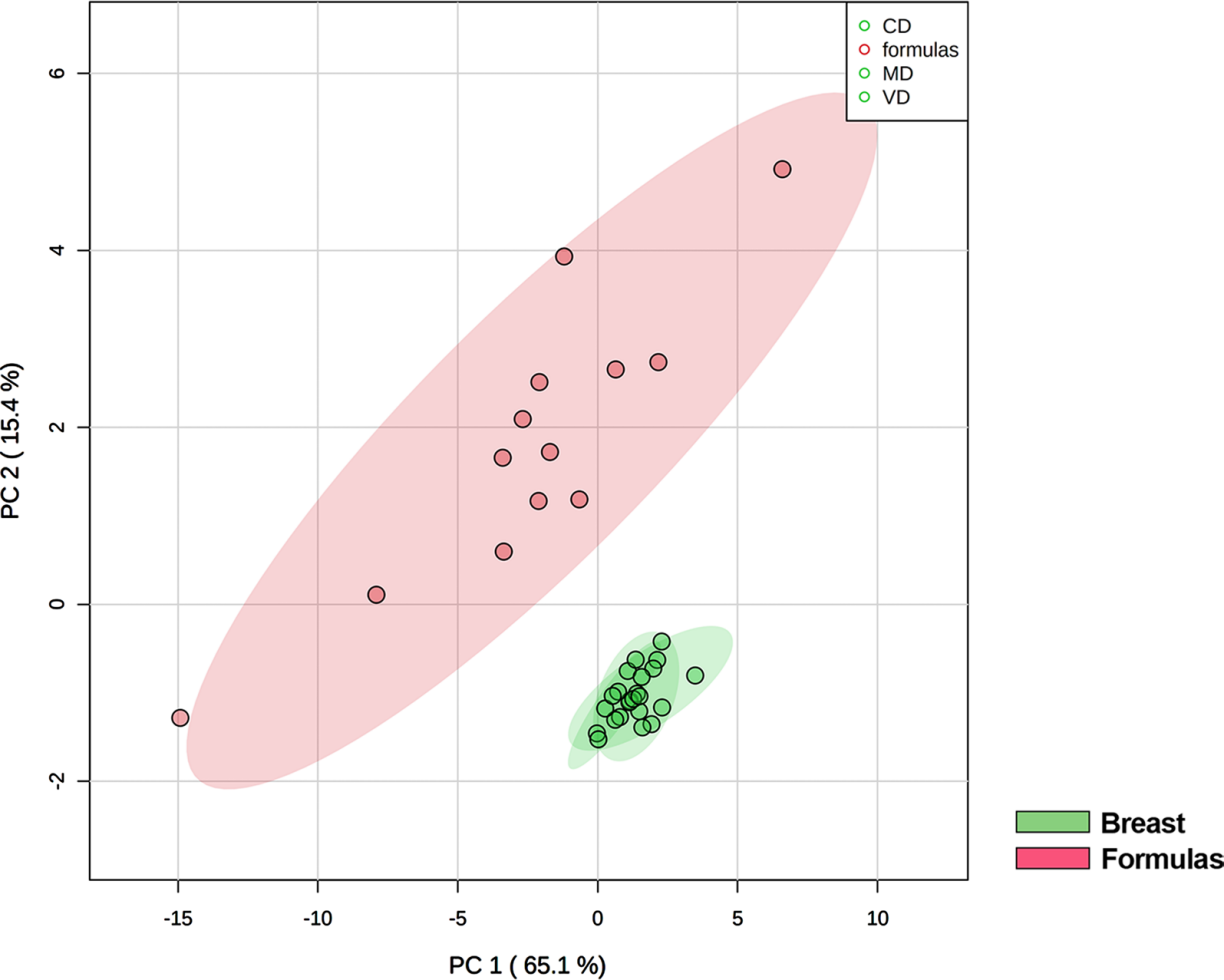
Comparison of commercial infant formulas (red) and breast milks (green) by PLSDA. The groups are separated on component 1 of 65%

Based on the data indicating no changes of HBM sphingolipid composition in relation to diet and gestational age, suggesting a specific sphingolipid metabolism in the mammary gland not easily influenced by external factors, we elected to study the sphingolipid composition of infant formulas (Fs). Thirtenn different Fs were considered and the results were compared with those of HBM. PLSDA analysis show that Fs have a sphingolipid composition very different from each other and truly distant from the composition of HBM (Fig. 5).

**Fig. 6:**
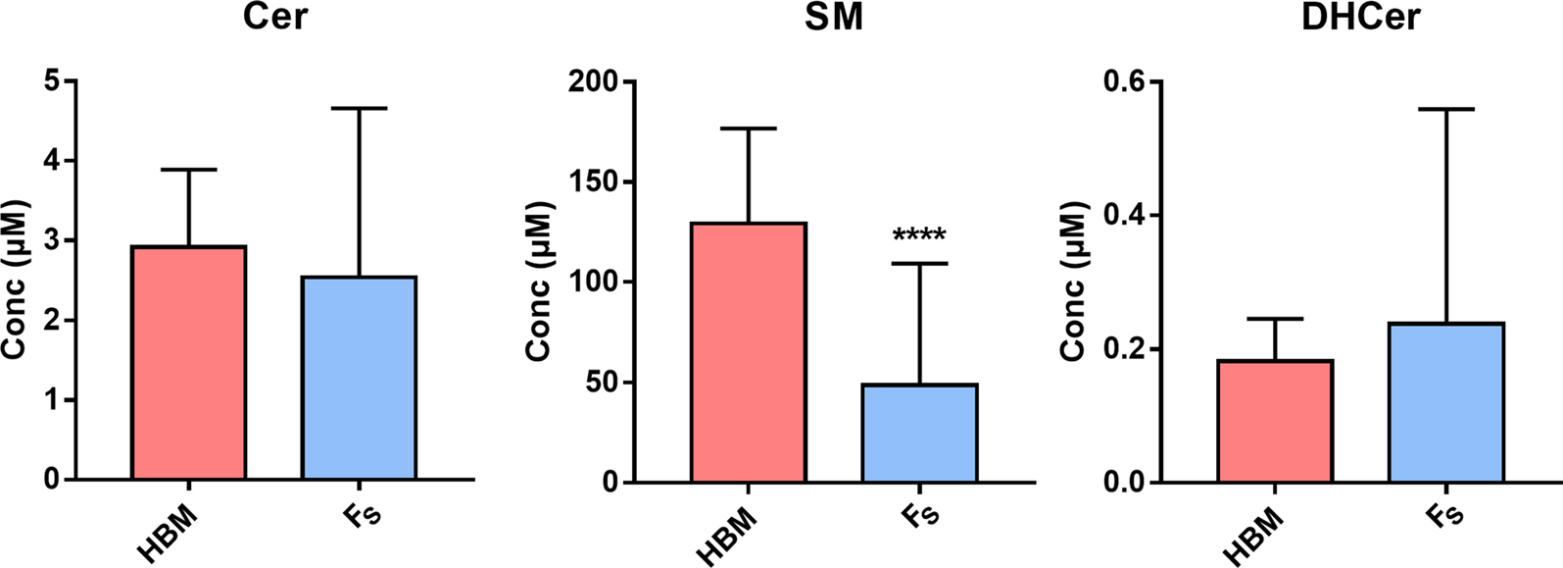
Sphs composition in infant formulas and human breast milk. Data are expressed as μM concentration and represent the mean ± SD of infant formulas (Fs, light blue, n = 12 excluding the outlier F9) and of the total human breast milks (HBM, red, n = 23) included in the study. Cer, ceramide; SM, sphingomyelin; DHCer, dihydroceramide. Statistical difference (**** p < 0.0001) was established by unpaired t-test

The PLSDA analysis evidenced among the Infant formulas an outlier sample (F9), which was therefore removed for the univariate analysis. By pooling all the Fs together and all the HBM together, the univariate analysis evidenced a considerable lower SM value. No statistical differences are present for Cer and DHCer (Fig. 6).

**Fig. 7:**
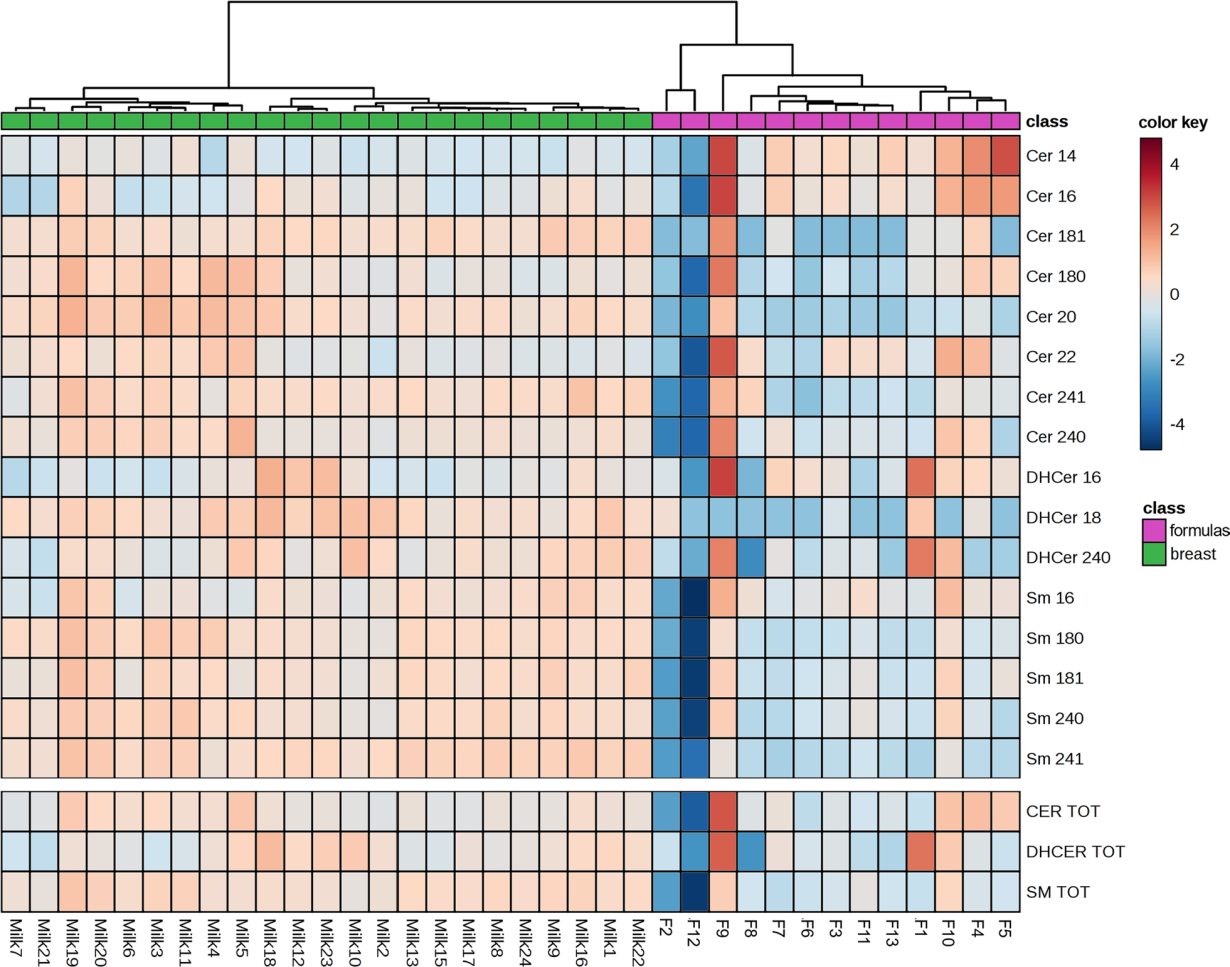
Hierarchical clustering coupled with heatmap representation of the Sphs in milk samples as function of the sources indicated as class: infant formulas (fuchsia) vs human breast milk (green). The concentrations were autoscaled and log-transformed for visualization. The color-scale differentiates values as high (red), mean (grey) and low (blue)

In Fig. 7 each sphingolipid species and total content in HBM samples are compared to those in each Fs samples through a heatmap.

It is evident a general enrichment of Sphs in HBM vs Fs, without significant differences between subjects. By contrast, the Fs are more heterogeneous and in general poorer in lipid content. However, of 13 Fs object of the study, 4 are specific for PTB infant. Notably, one F for PTB (F9) is highly enriched with Cers and DHCers and, in particular, with Cer14:0, Cer16:0, Cer22:0, and DHCer16:0. This F9 sample was specifically formulated for hypercaloric supplementation of pre-term infant under 1,5 kg. Two Fs for FTB infants have very low level of Sphs (F2 and F12). In particular, F2 show low level of Cers (especially C24:1 and 24:0) and SMs, and F12 of Cers, SMs, and DHCers (especially all SM species). Only one F (F1) contains a high level of DHCer (particularly C16:0 and 24:0). Moreover, F5 results rich in short chain Cer such as C14:0. Taking in account the remaining Fs, it can be seen that they are poorer in all SM species and in Cers with medium and long fatty acid chain (Cer18:1, Cer18:0, Cer20:0, Cer 24:1, Cer24:0) and richer in Cers with short chain fatty acids (Cer14:0, Cer16:0) than HBM (Fig. 7).

**Fig. 8:**
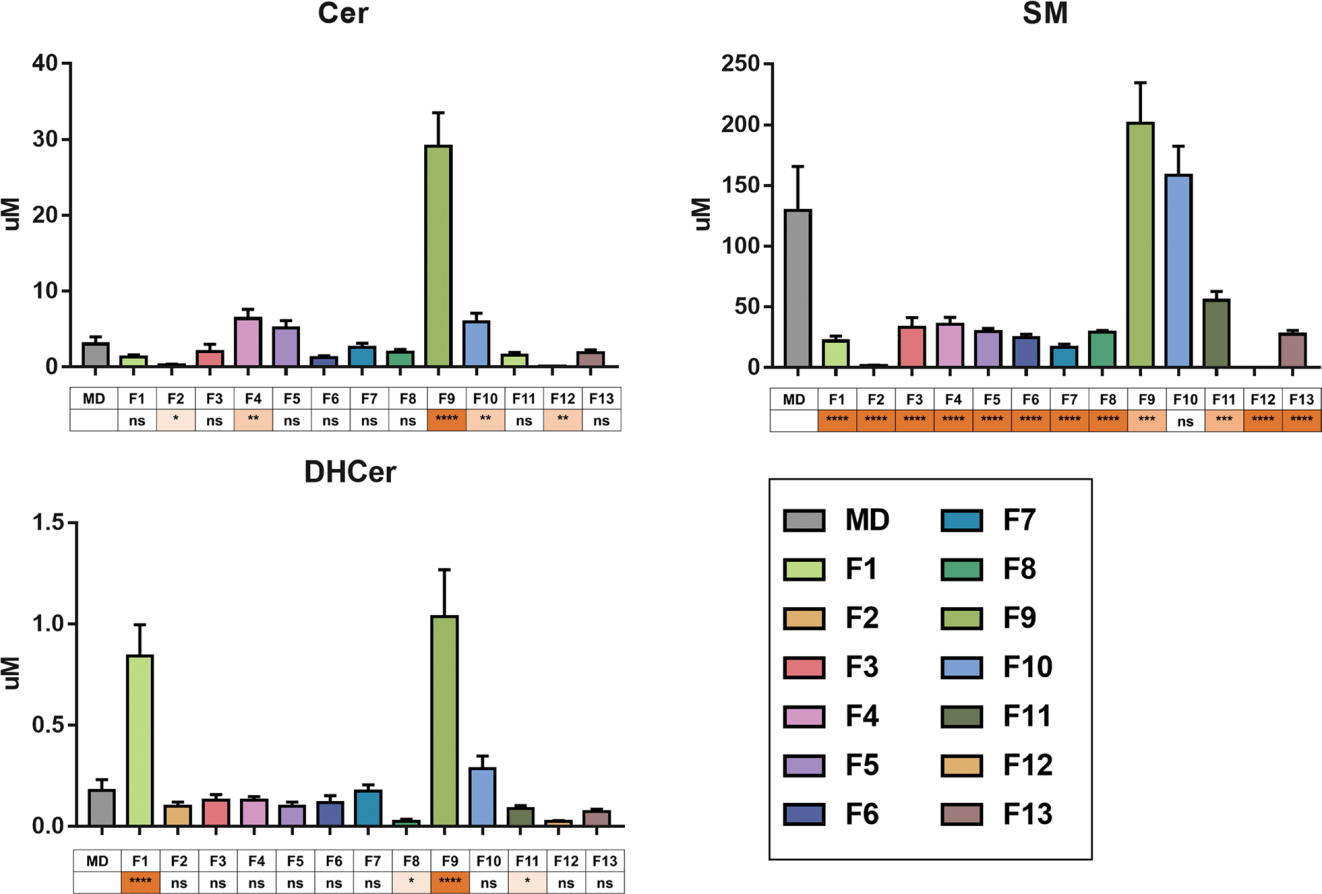
Sphs composition among different infant formulas (F, coded by different bars colours) compared with breast milk of mothers who followed the Mediterranean diet (MD, bars grey, n = 15). Statistical difference between groups (*p < 0.05; **p < 0.01; *** p < 0.001; ns, no statistical differences) was established by one-way ANOVA coupled with Bonferroni post hoc test against breast milk

Successively it was important to determine whether the differences between each Fs and the HBM of mothers who followed MD were statically significant (Fig. 8). Interestingly of 13 Fs included in the study, 11 have a SM content lower than HBM. In addition, the Cer content is lower in 7 Fs and higher in 4 Fs than in HBM. DHCer content is lower in 6 Fs and higher in 2 Fs than in HBM (Fig. 8).

As all the Fs included in the study are prepared from cow’s milk, to consolidate our study, we wanted to extend the lipidomic analysis to commercial cow’s milks consumed by adults. Thus, 4 whole and 2 semi-skimmed cow’s milks were considered.

**Fig. 9:**
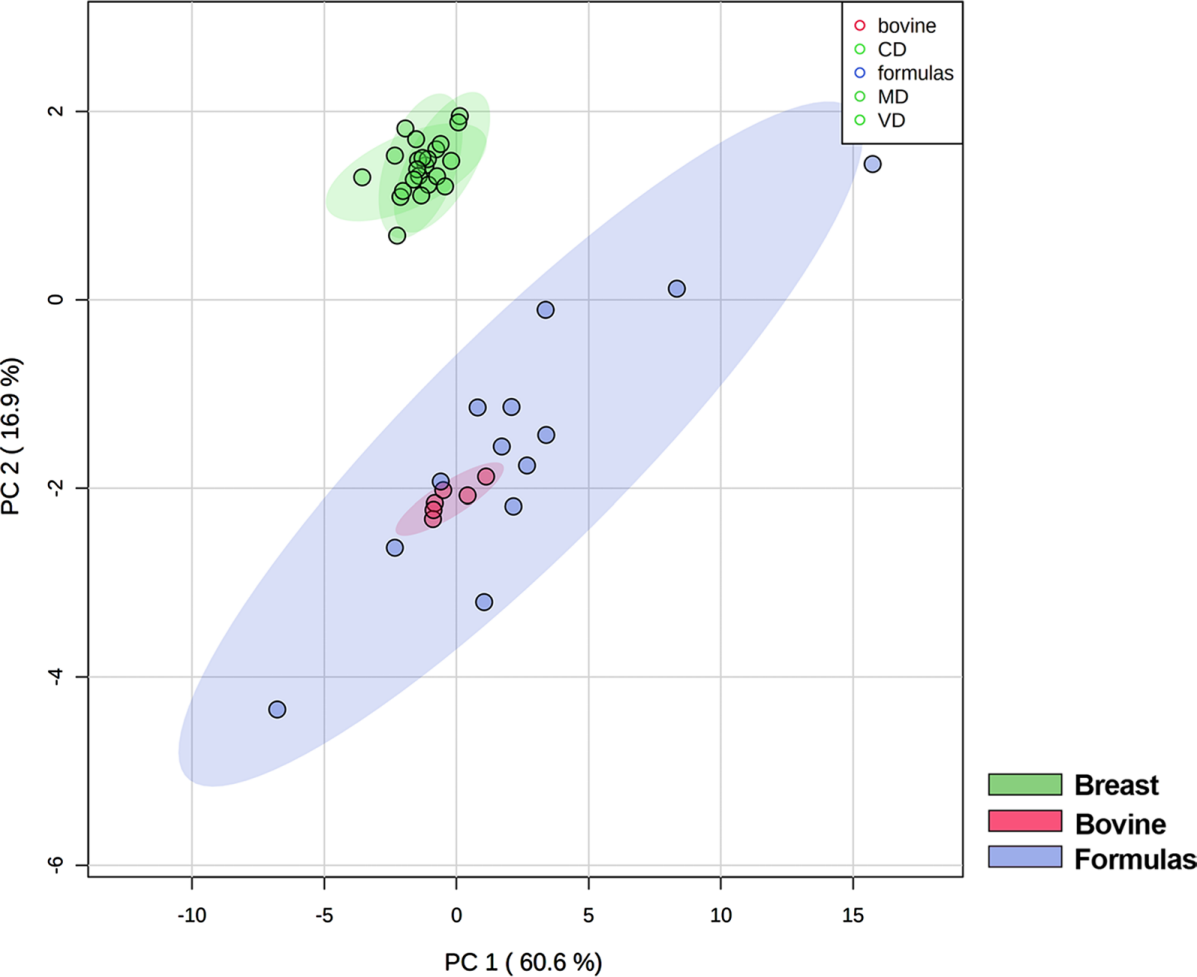
Comparison of commercial infant formulas, breast milks and cow’s milks by multivariate discriminant analysis (PLSDA). The groups are separated on component 1 of 61%

From PLSDA of Fig. 9, apparently the variations of Fs and adult milk compared to HBM seem to be similar, suggesting that Fs have characteristics similar to cow’s milk, as they are prepared from it.

**Fig. 10:**
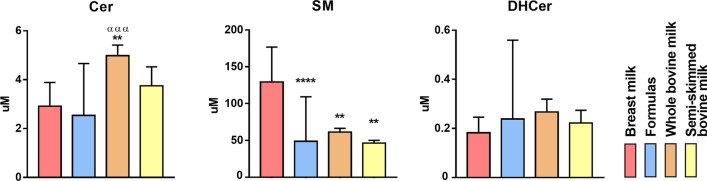
Sphs levels in different milk sources: human breast milk (coral, n = 23), infant formulas (pale blue, n = 12 excluding the outlier F9), whole bovine (orange, n = 4) and semi-skimmed bovine (pale yellow, n = 2) milks. Statistical difference was established by one-way ANOVA coupled with Bonferroni post hoc test * indicate statistical differences against the HBM, whereas α against infant formulas. (**p < 0.01; **** p < 0.0001; ^α α^ p < 0.01; ^α α α α^ p < 0.0001)

By univariate analysis, the amount of SM in both whole and semi-skimmed cow’s milks is less that in HBM, as well as in Fs. By contrast, Cer content is higher in whole milk compared to Fs (Fig. 10).

**Fig. 11:**
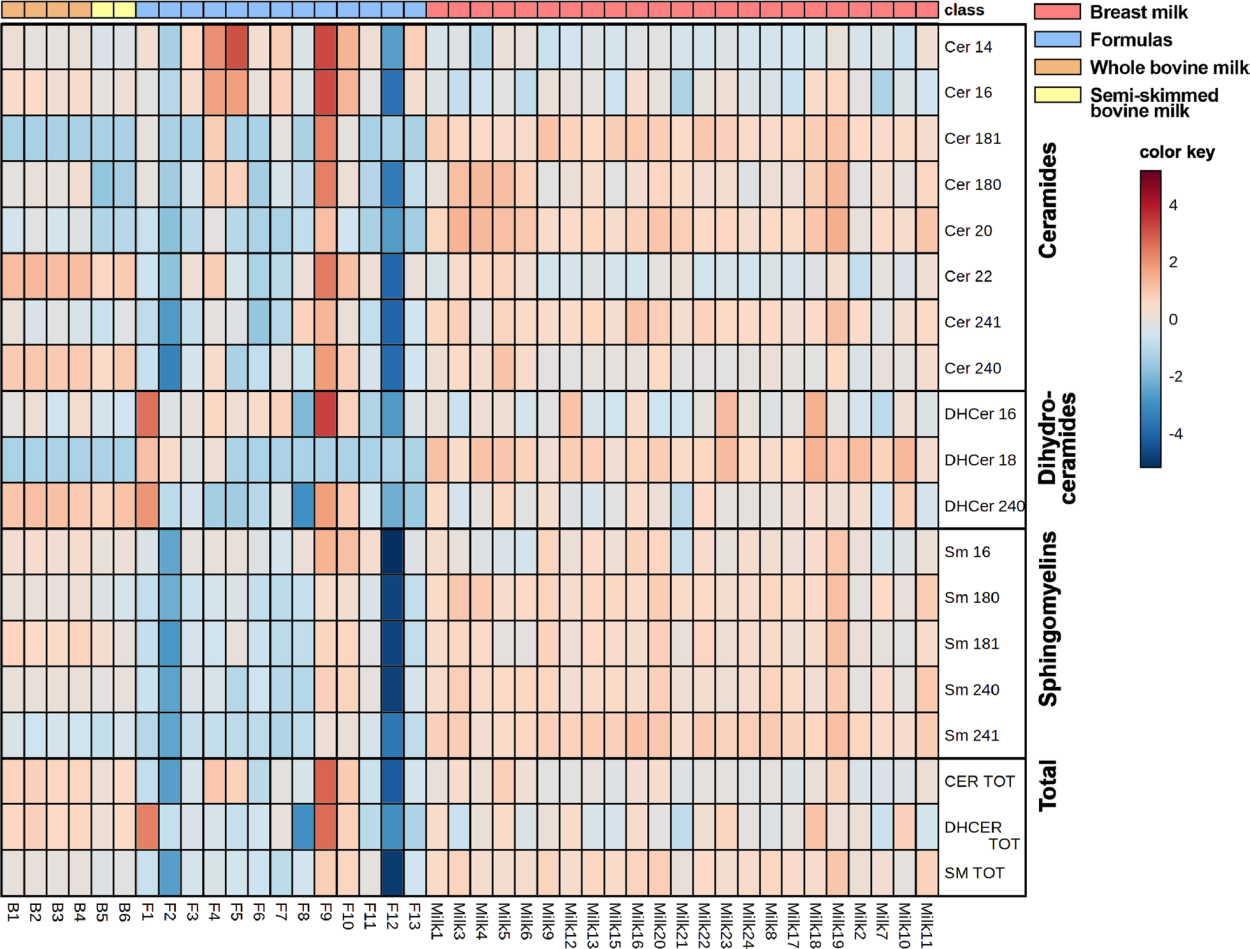
Heatmap representation of the Sphs concentration as function of the milk sources considered: HBM (coral), infant formulas (pale blue), whole (orange) and semi-skimmed (pale yellow) cow’s milks. The color-scale differentiates values as high (red), mean (grey) and low

In addition, analysing the sphingolipid species by heatmap, it is clear that some Fs undergo different modifications (F1, F2, F9, F12) compared to cow’s milks (Fig. 11). No important differences between whole (B1-B4) and semi-skimmed (B5, B6) milks were observed (Fig. 11).

## Discussion

### Mother’s eating habits and sphingolipids in breast milk

The relevance of Sphs in cell function and the essential role of diet in the physiopathology processes became decisive for us to investigate the Sph composition in HBM, recognized as the best source of infant nutrition. Our results highlight that SM, Cer, and DHCer contents and composition in HBM are not influenced by the diet that the mothers followed during pregnancy and breastfeeding. To our knowledge, this is the first study in which the composition of Sphs in relation to the mother’s eating habits is analyzed. It has been reported that humans digest and absorb most of the SM in normal diets and that the amount absorbed is influenced by realistic amounts of dietary SM [30]. We do not know exactly how much SM the mothers took with the diet but we do know that SM is present in 20-80 μmol/kg in fruits and vegetables, in 400-600 μmol/kg in in beef and chicken, and about 1.5-2.5 mmol/kg in dairy products, egg and soybeans [31]. Therefore, in our study, mothers who followed a MD or CD should have had a higher content of SM in milk than mothers who had followed VD. It is known that milk lipids originate by cytoplasmic lipid droplets at the apical plasma membrane of gland epithelial cells [32]. Thus, it is possible that the composition of the Sphs in HBM is linked to the specific metabolism of women’s mammary gland. In support of this hypothesis, our results show a very low variability of the sphingolipid milk composition in the 24 mothers, women with different weights, ages, and life habits. Ingvordsen Lindahl et al., 2019 [26] described significantly higher concentrations of SM, together with other polar lipids, in preterm milk respect to term milk. The difference was high in colostrum, reduced in transition milk and canceled in mature milk. We confirm that in mature milk no sphingolipid differences in relation to gestational age were found. An incomplete gestational period can affects the maturity of the mammary gland and its ability to secrete milk with the sphingolipid composition useful for the newborn’s condition. Therefore, nature has made sure that the mammary gland produces different amounts of Sphs in relation to the needs of the newborn and not in relation to maternal diet. At this point a question arises: why would sphingomyelin be so important for the newborn? The answer can be found in the literature data that demonstrate the importance of this lipid for the intestine health and for the maturation of the nervous system. In 2019 Milard et al. [33] showed the milk SM beneficial effects on intestinal functions through the stimulation of the intestinal tight junction expression mediated by IL-8. Norris et al. [34] described the protective properties of milk SM against dysfunctional lipid metabolism, gut dysbiosis, and inflammation. Regarding the effect of SM in the central nervous system, as early as 1999 Ledesma et al. reported that SM is involved in the myelination process and, therefore, in the axonal maturation [17]. Later Oshida et al. experienced the effect of dietary SM administration in developing rats at 8 days after birth demonstrating its positive effect on CNS myelination, a process closely associated with cognitive maturation [24]. It has been reported that lipid profile in embryonic hippocampal cells depends on nutrition factors [35]. In 2015 Henríquez-Henríquez et al. found that serum SM C16:0, C18:0, C18:1, C24:1, Cer C24:0, and deoxy-Cer C24:1 were significantly decreased in children affected by attention deficit-hyperactivity disorder [36].Then, in 2019 Schneider et al., after evaluating the levels of SM in infant nutrition products fed in the first 3 months of life, studied the cognitive development and myelination in neurotypical children, showing that a high level of SM was associated with higher rates of change in verbal development in the first 2 years of life, as well as with more prolonged rates of myelination in different brain areas [37]. From these data, the importance of the high content of SM in HBM is evident. There are no data in the literature on the content of Cer and DHCer in HBM. Our study for the first time pays attention that pays attention to these lipids. The results are relevant considering that Cer/DHCer level increases during rat brain development starting at embryonic stage until postnatal day and then gradually decreases until the maturity [38].

### Sphingolipids in human breast milk and in infant formulas

Interestingly, while the composition of breast milk is relatively stable regardless the mother’s diet, we found that the composition of the Fs is extremely variable, both in the content and in the composition of Sphs, and significantly different from HBM. We can speculate that the milk sphingolipid composition is species-specific: in fact, as shown in Figs. 9, 10 and 11, the sphingolipidome of commercial milk and Fs, both originating from bovine milk, seem to be similar, in particular in SM and DHCer content.We do not know at the moment the reason for the different composition of Sphs in HBM and bovine milk. We can only speculate that the baby has specific needs for SM for its growth and brain development. This hypothesis is supported by a study showing that of all the lipid classes of the rat brains fed HBM or IF, only SM was abundant in brains of HBM fed animals [39]. Therefore, it is possible to hypothesize that bovine newborn need low contents of SM as it has a complexity of the central nervous system lower than men's.

By contrast they are significantly different from HBM. Despite having the same origin, some Fs due to technological preparation processes are depleted or enriched in the content of SM and Cer species that make them extremely heterogeneous.

## Conclusions

In summary, our current study identifies SM, Cer/DHCer as molecules present in high concentration in HBM. This point out the importance of Sphs in baby nutrition for the brain development and cognitive functions. Since we found substantial differences with Fs, the next goal would be to produce formulas more and more similar to HBM.

## Supplementary information

**Additional file 1.** LC/MS-MS conditions for the analysis of ceramides, dihydroceramides and sphingomyelins.

## Data Availability

The authors confirm that the data supporting the findings of this study are available within the article.

## Abbreviations

Cer: Ceramide
DHCer: Dihydroceramide
GLS: Glycosphingolipid
HBM: Human breast milk
F: Infant formula
MD: Mediterranean diet
MF: Carnivorous diet
PT: Preterm birth
SM: Sphingomyelin
Sph: Sphingolipid
S1P: Sphingosine-1-phosphate
T: Term birth
VEG: Vegetarian diet
