# Supplementary material for: Human breast milk as source of sphingolipids for newborns: comparison with infant formulas and commercial cow’s milk
Source: J Transl Med. 2020 Dec 14;18:481. doi: 10.1186/s12967-020-02641-0 (PMC7734711; doi:10.1186/s12967-020-02641-0)
Supplement: Supplementary file 1 — Additional file 1. LC/MS-MS conditions for the analysis of ceramides, dihydroceramides and sphingomyelins. [file 12967_2020_2641_MOESM1_ESM.pdf]

Table X. LC/MS-MS conditions for the analysis of ceramides, dihydroceramides and sphingomyelins

**Analytical column**	ACQUITY UPLC BEH C8 1.7µm 2.1x100mm		
**Phase A**	0.2% formic acid 2 mM ammonium formate water-solution		
**Phase B**	methanol 0.2% formic acid 1 mM ammonium formate		
**LC gradient** (%B)	0-3 min (80-90%), 3-6 min (90%), 6-15 min (90-99%), 15-18 min (99%), 18-20 min (99-80%), 20-24 (80%)		
**Flow** (mL/min)	0.3		
**Column temperature** (°C)	30		
**Analytes**	**MS/MS** (m/z)	**DP** (eV)	**CE** (eV)
Cer 12:0 (IS)[1]	482.7 > 264.4	40	29.0
Cer 14:0	510.7 > 264.4	40	29.5
Cer 16:0	538.8 > 264.4	40	32.5
Cer 18:1	566.8 > 264.4	40	34.5
Cer 18:0	564.8 > 264.4	40	35.5
Cer 20:0	594.8 > 264.4	40	36.0
Cer 22:0	622.9 > 264.4	40	37.5
Cer 24:1	650.9 > 264.4	40	41.5
Cer 24:0	648.9 > 264.4	40	38.5
DHCer 16:0	540.4 > 266.4	40	33.0
DHCer 18:1	568.5 > 266.4	40	35.0
DHCer 18:0	566.5 > 266.4	40	35.0
DHCer 24:1	652.5 > 266.4	40	40.0
DHCer 24:0	650.5 > 266.4	40	38.5
Sm 12:0 (IS)[2]	649.6 > 184.1	40	50.0
Sm 16:0	705.6 > 184.1	40	50
Sm 18:0	733.6 > 184.1	40	50
Sm 18:1	731.6 > 184.1	40	50
Sm 24:0	817.6 > 184.1	40	50
Sm 24:1	815.6 > 184.1	40	50

[1] IS: internal standard for ceramides and dihydroceramides

[2] IS: internal standard for sphingomyelins
